# Pain Displays in Childbirth: How First-Stage Contractions are Interactionally Managed in Midwife-Led Births

**DOI:** 10.1080/08351813.2025.2450994

**Published:** 2025-02-21

**Authors:** Clare Jackson, Ann Weatherall, Victoria Land

**Affiliations:** a Department of Sociology, University of York, UK; b School of Psychology, University of Bedfordshire, UK; c School of Social Sciences and Humanities, Loughborough University, UK

## Abstract

Birthing is an exemplary setting for investigating how non-pathological painful sensations are intersubjectively established. Contractions are integral to giving birth and are physiologically normal events that can range from mild to intensely painful sensations. This conversation analytic study is the first to examine how first-stage labor contractions are made recognizable and shape interaction between laboring women, birth partners, and attending clinicians. Drawing on recordings from two British midwife-led units, we show how participants convey and recognize contraction pain through breathiness, pain cries, (limited) talk, and visible bodily actions. Contractions can be prospectively announced and/or retrospectively noticed. We demonstrate that breathing patterns become central to how participants collectively orient to and manage contractions, with the onset of pain temporarily suspending ongoing activities in favor of breath work. Data are in British English.

Labor pain presents a distinct context for studying how non-pathological pain arises in social interaction. Through conversation analysis (CA), we examine the interactional management of first-stage contractions, focusing on how these (variously) painful sensations are socially produced. After reviewing relevant literature on labor pain, multimodality, sensoriality, and CA approaches to pain (Sections “Labor contractions and pain”–“Multimodal resources for constituting pain sensation”), we present detailed analyses of (primarily) the onset of contraction sequences. While one contraction is shown in full (Section “A single contraction sequence”), the analysis focuses on how women display they are experiencing a contraction (Section “Laboring women announce a contraction”) and how others notice them (Section “Others recognizing the contraction”). We observe that the emergence of a contraction is omnirelevant for the parties and prompts a (temporary) suspension of ongoing talk and/or other clinical activities, which are recurrently replaced with a collective focus on the woman’s^1^^1^We acknowledge that not all people giving birth are women. We use the term because our laboring participants were so identified. breath (Section “Breathing, contractions and pain”).

## Labor contractions and pain

Pain during labor contractions is a complex multidimensional sensorial phenomenon (Lowe, 2002; Whitburn et al., 2019) encompassing physiological, psychological, social, and cultural dimensions (Labor & Maguire, 2008; Navarro-Prado et al., 2022). During the first stage of labor,^2^^2^In the biomedical model, physiological labor consists of three stages. First-stage uterine contractions open the cervix, second-stage contractions push the baby out, and third-stage contractions expel the placenta. pain arises from uterine contractions that cause cervical dilation (Melzack, 1993) and intensifies as they become stronger and more frequent (Lowe, 2002) across this stage. Contraction pain is physiological rather than pathological so, although potentially intense (Mathur et al., 2020), it also has a productive quality (Whitburn et al., 2017) marking progress toward birth.

The experience of contraction pain varies considerably (Lowe, 2002; Whitburn et al., 2019), shaped by psychological factors like anxiety and fear (Curzik & Jokic-Begic, 2011; Haines et al., 2012), social support (Bohren et al., 2019), and relationships with caregivers (Leap et al., 2010). These factors operate in specific historical, organizational, and cultural contexts that structure diverse understandings of birth (Jordan, 1992; McKinnon, 2016).

Thus, labor pain is simultaneously universal, cultural, private, and subjective. Like pain more generally, it is regarded as an intra-corporal experience that may escape adequate linguistic description (Scarry, 1985). Consequently, previous research has often treated pain as requiring communication from an inner experience to external observers. As Whitburn et al. (2019, p. 30) argued,

In order for an observer to “know” a person’s pain, the person must communicate it. They must transform that inner experience into words to describe it, and/or display “pain behaviours” that can be interpreted by an observer.

CA offers a different perspective by examining how pain is constituted and becomes relevant through observable conduct in interaction. However, there is a remarkable lack of research on *how* pain becomes relevant in the situated context of labor (Bergstrom et al., 2010 is an exception). Although there is relevant ethnographic research (e.g., Logsdon & Smith-Morris, 2017; Machin & Scamell, 1997), most qualitative studies have used retrospective accounts (e.g., Henrique et al., 2021; Lundgren & Dahlberg, 1998; Whitburn et al., 2017). Importantly, this research has centered women’s own descriptions in developing an experiential understanding of labor pain. Nevertheless, because labor (generally) occurs in an interactional context, rather than treating pain as a private sensation that must be communicated outward, we examine how pain becomes interactionally relevant through participants’ observable orientations to and coordination around specific practices. This ethnomethodological approach focuses on how pain is constituted as a social object through participants’ methods for displaying, recognizing, and responding to it.

To our knowledge, Bergstrom et al.’s (2010) study of the second (pushing) stage of labor is the only other analysis of labor pain in situated recordings (collected in the 1980s in the United States). Although not a CA article, Bergstrom et al. were concerned with women’s pain expressions, which were not always present, but might involve non-lexical forms (e.g., screaming), particularly through expulsive contractions, or lexical forms (e.g., cursing, demanding help) between contractions. Bergstrom et al. also noted various embodied actions associated with extreme pain or distress.

Our research focuses on the social production of the sensation of first-stage contractions in a contemporary British midwife-led setting, using CA to analyze how this sometimes intense but non-pathological pain shapes social interaction. We contribute to the growing literature on the multimodality of pain displays (e.g., Weatherall et al., 2021) and the broader area of sensoriality (e.g., Mondada, 2021). More specifically, because labor provides an exemplary setting for researching non-pathological pain, our study advances understanding of how “normal” and “productive” pain—a topic hitherto largely neglected in CA-is managed interactionally.

### Multimodality and sensoriality in interaction

The embodied turn in interactional studies (Nevile, 2015) contests language as a privileged mode of activity that acts independently of, and thus marginalizes, the body (Hofstetter et al., 2021). Recognizing the body as the material basis for generating social action, language is decentered and treated as one among multiple modalities, which include non-lexical sounds, bodily conduct, gaze, and object manipulation (Goodwin, 1979, 2000; Keevallik, 2018; Keevallik et al., 2023; Keevallik & Ogden, 2020; Mondada, 2014, 2016, 2021). These multimodal resources may form what Mondada (2024) termed multimodal gestalts or methodical practices for accomplishing action (e.g., a verbal request accompanied by pointing, gazing at and/or stepping toward an object). This holistic approach reconceptualizes conversation as involving parallel modalities, temporalities, and sequentialities (Mondada, 2016).

The body also mediates multiple modalities of sensation (e.g., seeing, hearing, tasting), traditionally regarded as inner individual processes (Mondada, 2021), raising questions about the limits of their effability. Interactional research now explores how people make sensations publicly available and accountable, including taste (Mondada, 2018a; Wiggins, 2002), smell (Mondada, 2020), and bodily strain (Hofstetter et al., 2021). These investigations have demonstrated that sensory experiences, far from being purely private phenomena, can be collaboratively constructed and negotiated in interaction (Mondada, 2021). Such insights inform our analysis of how laboring women make the pain sensations of contractions interactionally available to others.

### Multimodal resources for constituting pain sensation

Mondada (2021) noted that pain is often excluded from the traditional five-senses model, yet is it widely considered a subjective, private, and ineffable experience (Weatherall et al., 2021). However, given that pain is often associated with pathology (Kuner, 2010) and a common reason for seeking medical attention (Jordan et al., 2010), there is substantial literature on pain displays in clinical settings. In a foundational study, Heath (1989) found that pain was rarely expressed outside the doctor’s physical examination in primary care consultations (see also McArthur, 2018). Heath also observed a distinction between pain cries (e.g., sharp inbreaths and near-lexical expressions like *ooh*) and description, which can be combined so that displays of suffering might be filtered through words. These expressions are locally occasioned and organized to cooperate with doctors’ examinations/questioning rather than to attract attention to suffering *per se*. Thus, pain expressions tend to be led by doctors’ activities (McArthur, 2019) and when patient-initiated, occasion minimal responses from clinicians (Chapman & Beach, 2020). Galatolo and Fasulo (2022) demonstrated an interactional preference for minimizing pain in post-surgery amputee consultations and patients fitted their responses to the contingencies set up by doctors. Although pain is an inner individual experience, it is produced intersubjectively to serve locally produced and ratified interactional goals (McArthur, 2019).

Multimodal interactional resources, including non-lexical sounds, talk, and embodied actions, are central to intersubjectively establishing pain. Despite their phonetic diversity, non-lexical vocalizations achieve locally specific interactional projects (Keevallik & Ogden, 2020). In GP consultations, sounds like grunts, moans, and sharp intakes of breath constitute a flooding out of pain linked to an immediately prior event like a doctor’s touch (La & Weatherall, 2020; Weatherall et al., 2021). Audible breathiness, sudden intakes of breath, or breath-holding, along with grimacing and recoil, often mark the beginning of pain displays when a doctor touches a painful area, communicating immediate, embodied pain sensations and providing diagnostically useful information (Weatherall et al., 2021).

CA studies highlight breath’s interactional functions beyond mere speech production (Hofstetter et al., 2021; Ogden, 2024). Audible inbreaths, for instance, have been associated with incipient speakership (Schegloff, 1996) and can project multiunit turns (Rochet-Capellan & Fuchs, 2013). Breath can also constitute recognizable social actions (e.g., Hoey, 2014) and exhibit sensorial (Mondada, 2021) or shared physiological engagement (Pehkonen, 2020). As noted, in medical settings, breath plays a role in pain displays (Weatherall et al., 2021). In the context of labor, breath has additional significance as it is not only implicated in displaying pain but is also a key component in pain management techniques, both with and without respiratory pharmacological interventions like Entonox.

Building on analyses of multimodal pain displays in medical settings, we demonstrate how breathiness and other embodied actions (e.g., closing eyes and lowering the head) contribute to a multimodal gestalt (Mondada, 2024) establishing the onset of contractions. However, labor provides a distinctive context for studying pain in at least three ways. First, unlike the diagnostic focus of pain displays in primary care (Heath, 1989), labor pain is expected and even welcomed as a sign of progress. This fundamentally reshapes how pain is oriented to and managed by all parties. Rather than minimizing pain expressions (Galatolo & Fasulo, 2022) or treating them as diagnostically relevant (McArthur, 2018), midwives (and birth partners) treat pain displays as requiring active support. Second, in contrast to the short bouts of elicited pain typically examined in prior studies, the pain of labor contractions ebbs and flows over hours. Early labor features irregular contractions (every 5 to 15 minutes, lasting 30–60 seconds), becoming more regular and prolonged as cervical dilation increases (Lowe, 2002). This temporality requires sustained embodied and interactional practices for pain management. Finally, as neither the timing nor frequency of contractions are under the control of the laboring person, talk and clinical activities are necessarily interwoven around them. This start-stop temporal quality, combined with pain’s productive role in labor, creates unique conditions for studying how non-pathological pain is managed collaboratively in interaction. In this article, we show how these simultaneously physiological and social first-stage contractions are organized in interaction (see Mondada, 2021), with a particular focus on shared methods for displaying and understanding a painful contraction is occurring.

## Materials and method

The data stem from a corpus of consented recordings (24 video and 13 audio) of 37 women laboring in two midwife-led units in England, collected in 2018–2019 during an NIHR-funded project (Annandale et al., 2022). Each unit was provided with a mobile Smots^TM^ camera, and women could (request others to) position the camera, turn it on/off, and choose the recording format (audio/video). Recordings featured 43 birth partners and 74 healthcare professionals (primarily midwives, but also student midwives and obstetricians). Ethical approval was granted by the National Research Ethics Service Committee for Yorkshire and the Humber (no. 17/YH/0102).

Labor contractions were pervasive in the data. Initial identification of contraction sequences relied on broad criteria developed through familiarization with the data, including explicit verbal references to contractions and observable changes in the laboring woman’s conduct (e.g., posture, facial expressions, or breathing). Using CA methodology (Hoey & Kendrick, 2017), we selected recordings for detailed analysis. From our dataset, we excluded audio-only recordings (*n* = 13), videos with only second-stage+ contractions (*n* = 7), and those in which the laboring woman was off-camera (*n* = 3). From the remaining 15 recordings, we selected up to three contractions per recording with clear midwife-participant interactions, yielding 37 contractions for analysis. The final sample included eight first-time mothers; all had life-partners present, and two had additional family members.

Note that Entonox, a self-administered mixture of nitrous oxide and oxygen inhaled through a mouthpiece, is commonly used across our dataset. However, not all women used it for every contraction; therefore, our sub-collection includes cases with and without Entonox.

Each contraction was transcribed using Jeffersonian conventions (Jefferson, 2004) with an added superscript “e” to denote inbreaths taken with Entonox. We annotated the transcripts with a simplified adaptation of Mondada’s (2018b) principles to include embodied and multimodal features.^3^^3^Our extracts are too long for the detailed descriptions Mondada exemplifies working with shorter moments Where helpful and with consent, anonymized images accompany the extracts. Laboring women are identified in the transcripts by the first three letters of their pseudonyms, birth partners by BP, qualified midwives by M and a number indexing their appearance order in the recording (M1, M2, etc.), and student midwives by SM.

## Results

First, we show a single contraction in full (Section “A single contraction sequence”) to illustrate its interactional organization as it surfaces and recedes. We then examine the socially produced emergence of contractions, whether they are announced by the laboring woman (Section “Laboring women announce a contraction”) or recognized by others (Section “Others recognizing the contraction”). Finally, we demonstrate how parties collectively orient to breathing as fundamental to managing sensorial engagement with contraction pain (Section “Breathing, contractions and pain”).

### A single contraction sequence

In Extract 1, Phoebe, the laboring woman, announces a contraction’s onset and its subsidence is marked by the launch of a new action, namely, the midwife’s offer of a drink. The excerpt involves only two participants, because Phoebe’s birth partner is attending to car-parking. The midwife (M1) monitors the fetal heartrate using a Doppler ultrasound machine while Phoebe is in the pool. Uniquely in the dataset, Phoebe holds the Doppler head to her abdomen (typically, midwives apply the monitor). Phoebe had been using Entonox with contractions across the previous 50 minutes, but this was her first time using it in the pool. The extract begins a few seconds before monitoring is completed with a two-turn assessment sequence affirming Phoebe’s positive experience of the pool (lines 1–2).

**Figure:**
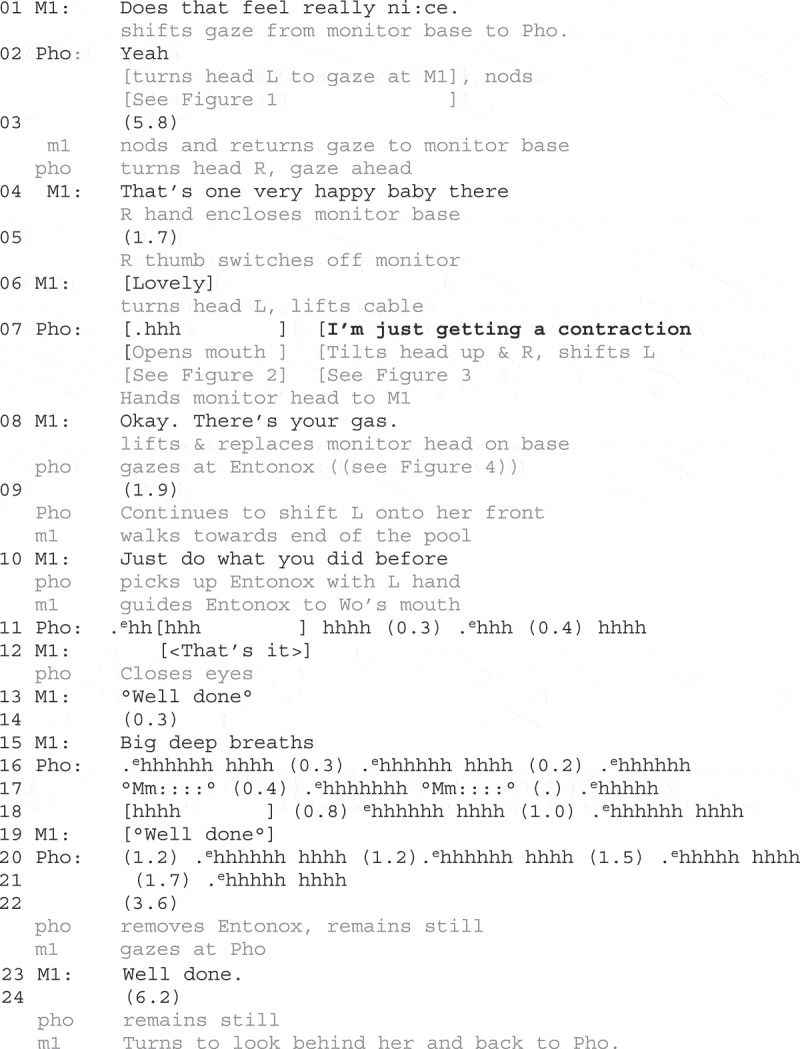

**Extract 1** [VIP36, Phoebe, Primip, 54:49]:^4^^4^Extract headings indicate: VIP (Voices in Partnership) study acronym, woman’s randomized participant number and pseudonym, primip (first labor) or multip (subsequent labor), and timestamp for extract start. “I’m just getting a contraction”

The contraction in Extract 1 emerges as monitoring draws to closure. M1 foreshadows this closure with her assessment that the baby is “very happy” and moving her hand to the monitor base (line 4) to switch it off (line 5), followed by a high-grade assessment “lovely” (line 6; see Antaki et al., 2000). Notwithstanding the weak conditional relevance of assessments (Stivers & Rossano, 2010) Phoebe passes an opportunity to respond to M1’s assessment in line 4. Instead, as M1 utters “lovely,” Phoebe opens her mouth to inhale (line 7; Figure 2), and then announces she is “just getting a contraction” (line 7), verbally marking its emergence.

**Figure:**
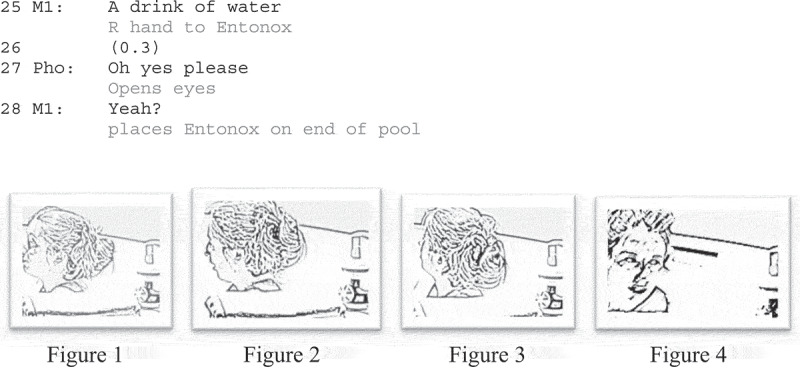

The sequential position of the announcement after passing a turn of talk, and an oral inhalation retrospectively renders those as resources projecting an emerging contraction. As Phoebe announces the contraction, she first tilts her head slightly up and to the right, away from M1 (line 7; Figure 3), then turns her whole body to the left, handing the Doppler head to M1 (line 7). She turns and gazes at the Entonox (line 8; Figure 4) on the side of the pool, continuing until she lies on her front (line 9) and can pull it into her mouth (line 10). The verbal announcement and the embodied actions together form what we suggest is a multimodal gestalt of an emerging contraction. Phoebe’s turn to the Entonox treats the surfacing contraction as requiring pain management.

Upon acknowledging Phoebe’s contraction announcement (“okay,” line 8), M1 anticipates, and cooperates with immediate pain management by indicating the Entonox (“there’s your gas”; line 8). Using the possessive pronoun “your” treats Entonox use as established for this participant (see Tennent & Weatherall, 2024) and implies Phoebe’s ability to self-administer it. After placing the Doppler head on the base, M1 walks to the end of the pool to assist Phoebe with the Entonox, instructing her based on their shared previous experience: “just do what you did before” (line 10).

Phoebe closes her eyes when she starts inhaling Entonox (line 11), marking a shift in the participation framework from engaged interaction to focused engagement with breathing. She keeps her eyes closed for the full period of Entonox use and for some seconds afterward (i.e., lines 11–27). Her 13 Entonox inhalations (lines 11, 16–18, and 20–21) are consistently timed and rhythmic with regular intervals between inbreaths. Generally, Phoebe’s outbreaths are non-vocal, but on the fifth and sixth, she emits quiet but discernible non-lexicalized bi-labial nasal sounds (“mm:::,” line 17). These vocalized breaths, positioned midway through the contraction sequence, constitute, and make socially available, a peaking of sensation from the contraction.

While Phoebe contracts, M1 affirms and sustains what Phoebe is doing, using actions with little response relevance but which configure Phoebe’s contraction as requiring guidance. For example, M1 says, “that’s it” during the first inhalation of Entonox (line 12), thereafter making positive assessments (“well done,” lines 13 and 19) and issuing instruction (“big deep breaths,” line 15). M1’s assessments and instructions foreground breathing in the use of Entonox. Except for the overlap between Phoebe’s inhalation and M1’s “that’s it,” M1’s utterances occur between inhalations either in overlap with an outbreath (line 19) or in the gap between an outbreath and following inbreath (lines 13 and 15), suggesting she generally times her utterances between Phoebe’s inhalations. The position of M1’s turns effectively constructs a local temporal order in which Phoebe’s inbreaths are oriented to as turn units. Berducci (2016) documented an analogous practice with infant crying. The fine temporal tuning of instructions with others’ embodied actions has also been established (Simone & Galatolo, 2021).

The sequence’s completion is interactionally achieved through several coordinated moves. Phoebe marks the end of her need for pain relief by removing the Entonox from her mouth (line 22), but she keeps her eyes closed, a configuration that M1 treats as transitional. M1 orients to this shift by initiating the first action since the breathing sequence began that makes response relevant: a declarative offer of water (line 25; Entonox dries the mouth). Simultaneously, M1 moves to take the Entonox away from Phoebe, showing her understanding that the prior activity is complete. After a small delay (line 26), Phoebe opens her eyes and accepts the offer (line 27). Phoebe’s response is preferred albeit delayed, progressing the initiated action by taking the water.

In summary, this single, short contraction sequence demonstrates a cluster of embodied features, including an inbreath and a shift in head orientation, that form a multimodal gestalt establishing its emergence, which is also verbally attributed via Phoebe’s announcement. The coordinated actions around obtaining Entonox-Phoebe’s reaching and M1’s anticipatory assistance-demonstrate their shared orientation to the local relevance of pain management. Breathing, in the above case with Entonox, marks a period of intense sensation, and the organization of the peak is a shift to non-lexicalized vocalizations interpolated into breaths. During the period of primary focus on breathing, M1 orients to Phoebe as available primarily as a hearing recipient. The shift in participation framework out of and into engaged interaction as the contraction emerges and subsides suggests contractions have a distinct temporal organization relative to mundane social interaction, a matter that is more strongly established in the next sections.

### Laboring women announce a contraction

The next excerpts, all examples of the laboring woman announcing a contraction, provide further evidence of how the beginnings of contractions are organized. Broadly, announcements introduce new, contextually relevant information (Terasaki, 1976) and often appear in both ordinary and institutional interaction as part of news-telling sequences (e.g., Maynard, 2003) and may project extended turns of talk. However, in our data, while sharing the sense of “news,” the announcements project a shift to primary engagement with the contraction through the suspension of ongoing talk. The first examples in this section are clearest in terms of how the contractions organize the progressivity of turns and sequences of talk. The later examples were selected for their brevity and clarity in demonstrating participants’ collective orientation to contractions as taking precedence over routine clinical activities.

In Extract 2, a student midwife (SM) enters the room and initiates a personal state inquiry (line 1). Claudia establishes the possible beginning of a contraction by reaching for Entonox (line 2; Figure 5). Hence, both the immanence of a contraction and the response to SM’s question are relevant for Claudia. Her responsive turn (line 3) includes the announcement of an impending contraction.

**Figure:**
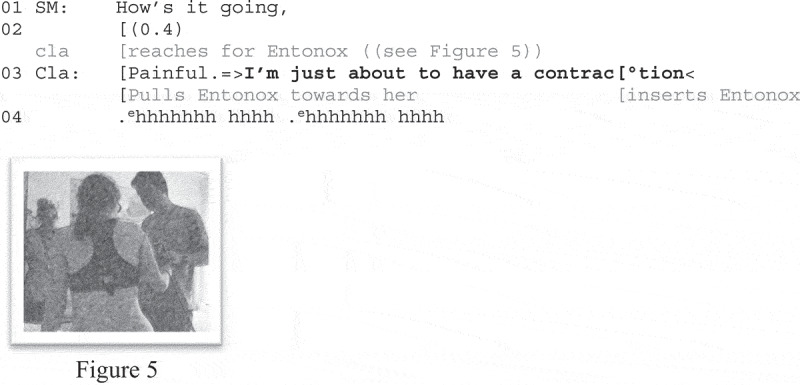

**Extract 2** [VIP22, Claudia, Primip, 06:03:24]: “I’m just about to have a contraction”

In the above extract, the verbal response to “how’s it going” is a simple, “painful.” However, this is delayed as Claudia reaches for Entonox (Figure 5), a movement that conveys the emergence of a contraction, which could also serve as a multimodal response to SM’s question. The verbal response is trouble-premonitory, potentially inviting further discussion (Jefferson, 1980). However, in the extension of Claudia’s turn, the transition relevance space between the first turn-constructional unit and the next one is compressed for a contraction announcement (“I’m just about to have a contraction”; line 2). The articulation of the word “contraction” is compromised by the insertion of the mouthpiece, suggesting a rush to use Entonox to manage the emerging pain, which she does next (line 4).

In Extract 2 Claudia’s actions are organized with respect to both sequence and the emergent contraction. In Extract 3, a contraction emerges into an ongoing sequence initiated when Kay asks about her options for pain relief (“What are other possibilities are there of uhm like pain relief and stuff”; lines 1–2). Pain is thus relevant throughout this extract: first as a topic of discussion; then as a displayed embodied phenomenon, which is oriented to as taking priority over progressing the sequence; and, finally, as a resumed topic once the pain display subsides.

**Figure:**
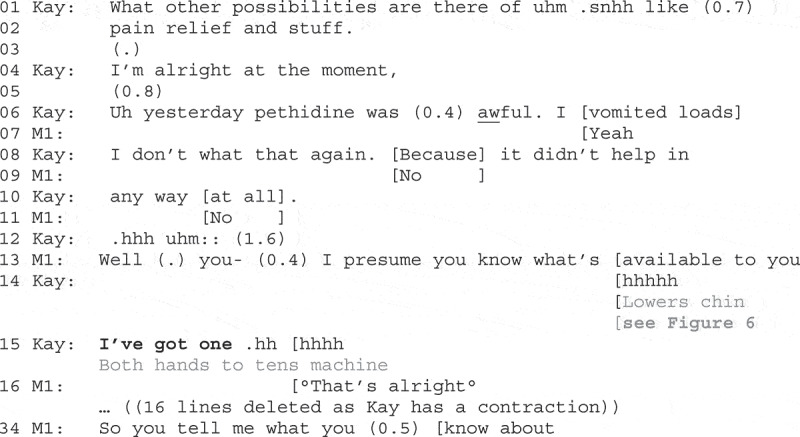

**Extract 3** [VIP06, Kay, Primip, 04:07:45]: “I’ve got one”

**Figure:**
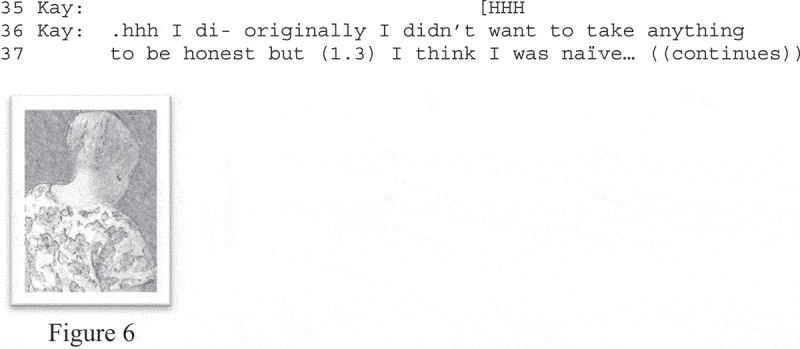

To avoid a potentially projected misaligned response (note M1’s silence in line 3) to her initial query (lines 1–2) about “possibilities” for pain relief, Kay adds to her turn, clarifying that she is not requesting it now (line 4) and explaining a previous negative experience with opiates (lines 6–10). Up to line 13, M1 has not provided a relevant answer to Kay’s initial question. At line 13, M1 begins to respond with a well-prefaced turn, which Heritage (2015) established functions as an alert that the turn it precedes will privilege its speaker’s perspective. In this case, it serves to resist providing an answer, based on the expressed assumption that Kay already knows the requested information.

As this sequence unfolds, new priorities emerge. In overlap with M1’s well-prefaced turn, Kay produces embodied conduct-lowering her chin (Figure 6) and exhaling (line 14). The significance of these actions is adumbrated when Kay announces that she has “got” a contraction (line 3), and moves both hands to her TENS machine, which provides a form of pain relief. Like Phoebe and Claudia’s reaching for Entonox, Kay demonstrates an immediate orientation to pain management. M1 orients to this new priority through her receipt (“That’s alright,” line 4) and further talk is suspended. The temporary nature of this suspension becomes clear when M1 later resumes the pain relief discussion with a counter request: “so you tell me what you know about” (line 34). Launching this request also shows M1’s orientation to the subsidence of the contraction. Kay responds to the counter request, showing for her, too, the contraction is no longer the primary organizing force in the interaction.

Extracts 2 and 3 show how the emergence of contractions shapes the progression of sequences of talk. Announcements may also function to suspend the progression of routine clinical activities, evidenced in the next two extracts. In Extract 4, a midwife (M1) is preparing to give Harmony an anti-sickness injection. Harmony asks her to wait, explaining she has “got a contraction” (line 1).

**Figure:**
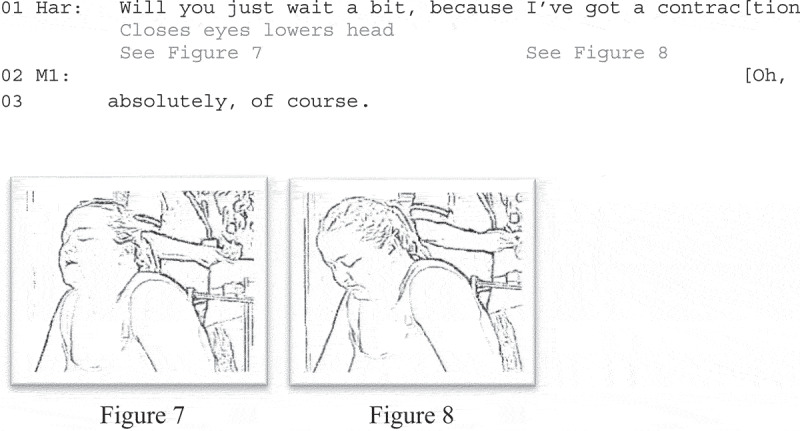

**Extract 4** [VIP30, Harmony, Primip, 28:09]: “I’ve got a contraction”

Harmony’s announcement and request work together to constitute the relevance of postponing the injection. As she speaks, she produces embodied conduct that marks an emerging contraction-closing her eyes (Figure 7) and lowering her head (Figure 8)-and a shift toward primary engagement with it. Notably, M1 grants the request before its completion. Further, M1’s oh-prefaced response, (lines 2–3) treats Harmony’s request as inapposite (Heritage, 1998), whereas her emphatic agreement (“absolutely, of course”) displays her understanding that the suspension of clinical activities during contractions is taken-for-granted in this setting.

In Extract 4, a contraction suspends the progress of giving an injection; in Extract 5, it suspends routine monitoring of fetal heartrate, which the midwife (M3) had signaled by switching on the Doppler machine before the start of the extract (not shown).

**Figure:**
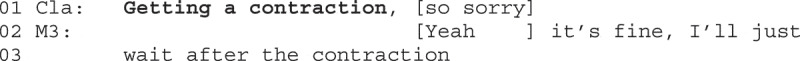

**Extract 5** [VIP22, Claudia, Primip, 06:38:10]: “Getting a contraction”

Claudia’s announcement that she is “getting a contraction” (line 1) implies she will be unavailable for fetal heartrate monitoring. The upgraded apology at the end of her turn orients to the interruption as a transgression. M3 displays her understanding by accepting that a contraction is under way “yeah” and indicating it not to be a problem with “that’s fine” (line 2) and that she’ll “just wait” (lines 2–3).

In summary, these extracts show how participants orient to and constitute pain displays as occasions for suspending ongoing activities. Through verbal announcements, embodied conduct, and coordinated responses, participants collectively establish the precedence of contractions over other interactional projects. The next extracts further detail these practices, focusing on how co-present others recognize and respond to emerging pain displays.

### Others recognizing the contraction

Midwives’ recognition of an upcoming contraction is a form of professional vision (Goodwin, 1994). Although midwives routinely demonstrate such vision (Extracts 6 and 7), birth partners may display similar competence (Extract 8), suggesting these practices can be acquired through sustained participation in the birth room.

**Figure:**
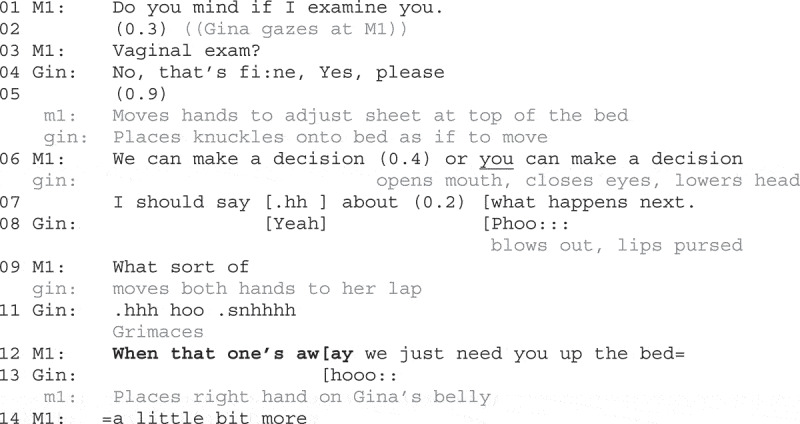

**Figure:**
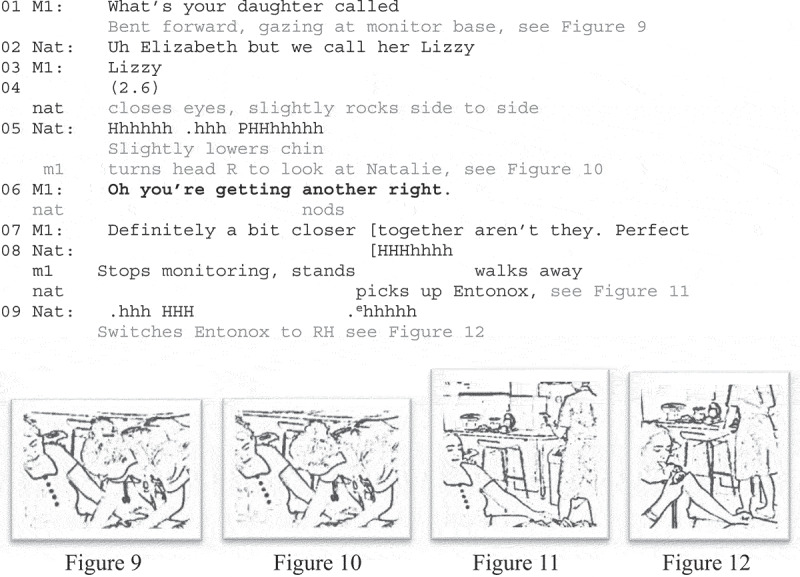

**Figure:**
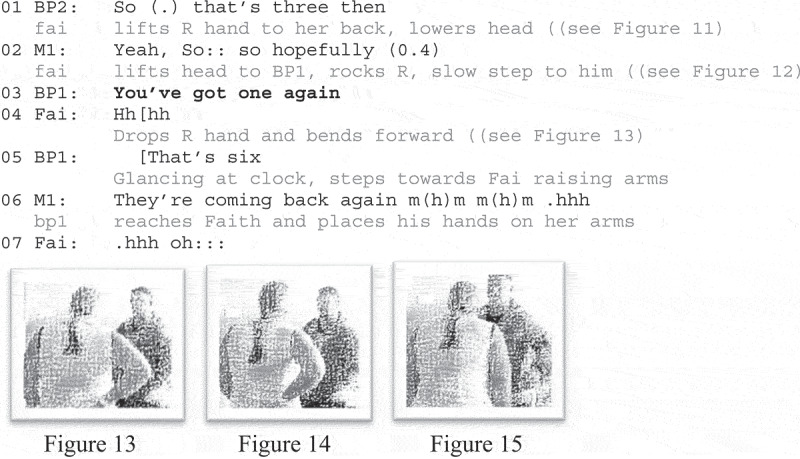

Extract 6 shows how this noticing is accomplished during a preparation for a routine clinical activity. As Gina sits on the side of the bed, M1 initiates a sequence by requesting to conduct a vaginal examination (lines 1 and 3).

**Extract 6** [VIP23, Gina, Primip, 01:58:15]: “When that one’s away”

Following Gina’s agreement to the vaginal exam (line 4), both she and M1 initially orient to its projected course. Gina positions her hands to assist movement up the bed, and M1 adjusts the sheet, likely preparing for Gina to lie down (line 5). M1 then indicates there is a joint decision to be made, repaired to Gina’s decision (lines 6–7), informed by the outcome of the examination. During M1’s repair, Gina produces a cluster of embodied actions: opening her mouth, closing her eyes, and lowering her head. While maintaining minimal engagement with M1’s turn through “yeah” (line 8), she then produces an extended voiced exhalation “Phoo:::.” M1 treats this multimodal gestalt as constituting an emergent contraction by halting her in-progress turn (“what sort of,” line 9; note also Gina’s retraction of hands to her lap), before explicitly tying the progression of the examination to its completion: “when that one’s away, we just need you up the bed a little bit more” (lines 12 and 14).

Extract 7 also illustrates a midwife’s noticing an emergent contraction and subsequently abandoning an ongoing activity. As the extract begins, the midwife (M1) is monitoring the fetal heartrate and she asks Natalie for the name of her elder child (line 1). Natalie is sitting on a birth ball facing the bed (Figure 9).

**Extract 7** [VIP32, Natalie, Multip, 59:10]: “you’re getting another”

The adjacency pair sequence at the outset shows Natalie is an engaged participant. However, she passes the opportunity to confirm her daughter’s shortened name following M1’s post-expansion repeat (line 3; see Aldrup, 2023) and in the subsequent silence (line 4), Natalie closes her eyes and begins to slightly rock on the birth ball. Then she audibly exhales and inhales (line 5), which appears to attract M1’s visual attention (head turn to Natalie; Figure 10). M1 explicitly acknowledges the onset of “another” contraction, treating them as a recurrent phenomenon (line 6). This is oh-prefaced, marking a change in epistemic status that can be sourced to prior information (Heritage, 1984), here, Natalie’s audible breathiness. Natalie confirms with a head nod (line 6). The midwife then approvingly remarks that the contractions are more frequent (line 7). Over this turn the midwife stops monitoring, stands up and walks away to a countertop at the back of the room (Figure 11). Although exogenous to the data, we know that NICE Guidelines (2014) recommend at least one minute of monitoring. At the point M1 halts the activity, she had been monitoring for about half this time, so the stopping is “early” and apparently motivated by her noticing the contraction and Natalie’s confirmation.

Extracts 6 and 7 illustrate midwives recognizing a contraction. Extract 8 demonstrates a more unusual case of it being done by a birth partner. Faith is attended by two birth partners: her life-partner (BP1) and her mother (BP2). The extract opens with Faith mother’s calculation of the number of contractions over a recent period (“so that’s three then”; line 1).

**Extract 8** [VIP15, Faith, Primip, 04:59:03]; “You’ve got one again”

As her mother speaks, Faith lowers her head and places a hand on her back (Figure 13). Responding to the mother, the midwife (M1) confirms (line 2) and begins but then abandons a new turn-constructional unit that is heading for an optimistic observation of the frequency of contractions (possibly completed in line 6). As M1 speaks, Faith lifts her head toward her partner (BP1) and takes a slow step toward him (Figure 14). M1’s abandonment of her turn is likely an orientation to Faith’s movements, but it is BP1 who makes it verbally explicit: “You’ve got one again” (line 3). BP1 treats Faith’s breathiness and embodied actions in line 4 as confirmation of the onset of a contraction with his comment “that’s six,” which is an attribution of the number of minutes since the last contraction (line 5).

Extract 8 is a good example of the relevance of contractions to the parties in the interaction. BP2 is counting them and BP1 is tracking the intervals between them. While progression of labor is omnirelevant, the birth partners’ actions have particular significance here because the midwife has previously expressed doubt about whether Faith’s contractions are sufficiently frequent to warrant her presence on the unit. Thus, commentary on number and frequency of contractions is available for the overhearing midwife, who concedes that “they’re coming back again” (line 6).

Across the extracts analyzed so far, we have shown several multimodal resources for constituting an emerging contraction. Laboring women may close their eyes (Extracts 1, 4, and 6–7) and/or otherwise display they are withdrawing from engaged interaction to manage the sensation (e.g., by lowering their head; Extracts 3–4, 6–7,) and/or reaching for pain relief (Extracts 1–3). They might announce a contraction (Extracts 1–5), but if they do not, others may acknowledge its (possible) emergence (Extracts 6–8). Breathiness is a recurrent feature of onset, which we next consider in more detail.

### Breathing, contractions and pain

Audible breathiness recurs with contractions, taking various forms, including Phoebe’s open-mouthed inhalation (Extract 1), Kay’s extended outbreath (Extract 3), and Gina’s voiced pain cry outbreath (“phoo”; Extract 6). Furthermore, participants retrospectively and intersubjectively recognize breathiness as marking the onset and progression of pain displays. Thus, breathing during labor is accountable in the ethnomethodological sense of the term as both ordinary and normative (Robinson, 2016).

Literature on birthing generally supports the benefits of using slow, deep breathing without Entonox to alleviate labor pain (Smith et al., 2018). Entonox is a form of pain relief in its own right but relies on breath for its delivery, and midwives recurrently refer to breaths during its use (e.g., Extracts 1 and 9). In what follows we further detail how breath becomes an interactional focus.

**Figure:**
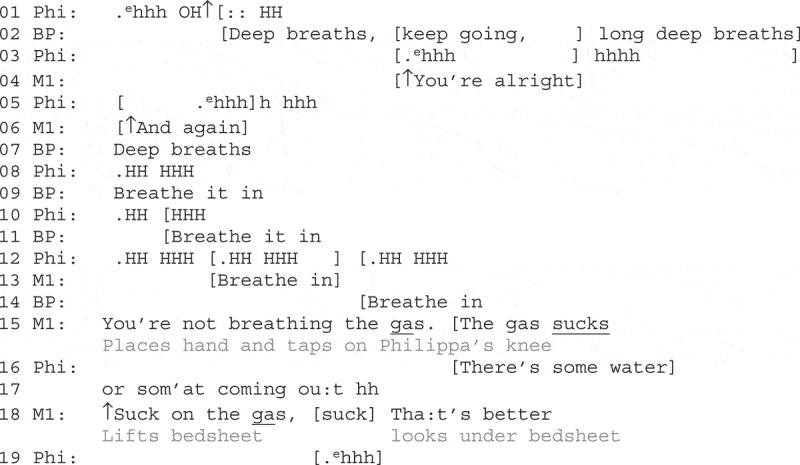

The normative character of breathing Entonox is clearly shown in Extract 9. We join the interaction as a contraction is under way and Phillipa is using Entonox (lines 1, 3, and 5). Her pain cry (line 1) occasions the birth partner and the midwife to offer breathing instructions and reassurance (lines 2 and 4).

**Extract 9** [VIP11, Philippa, Multip, 41:19]: “Breathe it in”

Philippa stops using Entonox (end of line 5) but continues to display a contraction via her audibly short and fast breathing (lines 8, 10, and 12) while continuing to hold the mask in her mouth, with her eyes closed. The birth partner and midwife (M1) orient to the relevance of the contraction by issuing directives to inhale the Entonox-“Breathe it in” (lines 9 and 11), then in a more truncated form: “Breathe in” (lines 13–14). M1 identifies a problem with Philippa’s breathing, shown by her negative observation (“you’re not breathing the gas”; line 15) at the same time as tapping on Philippa’s knee. Tapping acts as a multimodal summons (Routarinne et al., 2020), whereas the negative observation conveys that inhaling the Entonox is relevantly missing. In the expansion to this turn, M1 explains the appropriate use of Entonox– “the gas sucks.” Following Philippa’s troubles-report regarding a sensation of fluid loss (lines 16–17), and further directives to suck (line 18), Philippa reengages with the Entonox, which occasions M1’s positive assessment (“that’s better”; line 18).

There can be a focus on breath even when Entonox is not being used, as illustrated in Extract 10. This continues from Extract 8, and we rejoin the interaction as Faith lifts her hands to BP1’s shoulders (Figure 16) and issues pain cries (lines 7–10).

**Figure:**
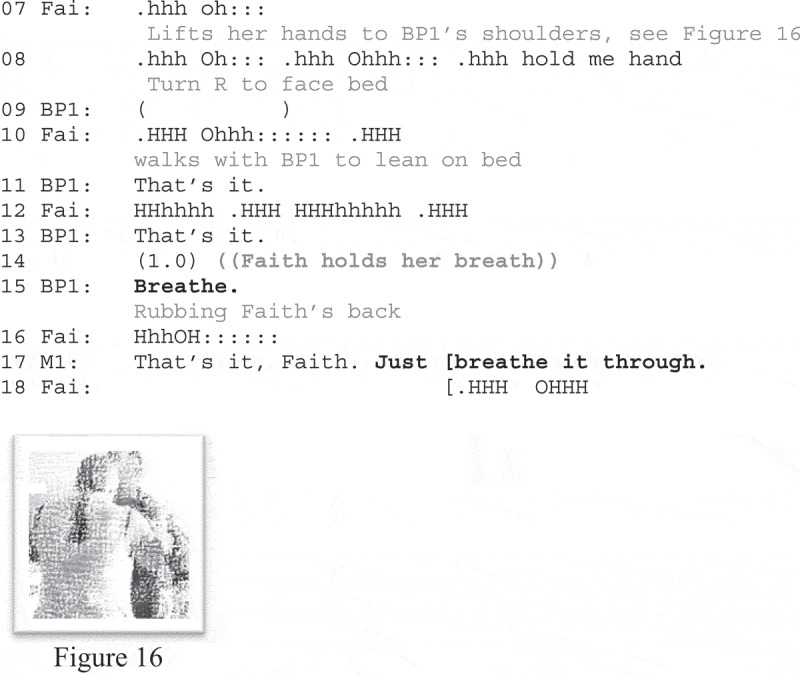

**Extract 10** [VIP15, Faith, Primip, 04:59:17]: “Breathe”

Together with embodied actions, Faith’s ongoing display of a contraction is audibly conveyed through her breath. Her inbreaths get louder (line 10) and her outbreaths are frequently voiced (“oh”; lines 7–8, 10, 16, and 18). In line 14, Faith visibly holds an inbreath (taken at line 12), occasioning BP1’s instruction to “breathe” (line 15). Following Faith’s release of held breath (line 16), the midwife positively receipts it, “That’s it, Faith” (line 17), and further directs her to “just breathe it through.”

Extract 11 also shows a focus on breath. Just before this extract starts, the midwife suspended an agreed activity to palpate Elise’s abdomen to establish fetal position when Elise had conveyed pain. However, at that point, Elise claimed not to know whether to call her subjective experience a contraction but continued to display pain. Noticing this, the midwife shifted from standing preparatory to the abdominal exam to sitting on the bed (data not shown, but see Figure 17). We join the interaction as Elise breathes quickly and loudly before describing her pain: “it hurts” (line 1).

**Figure:**
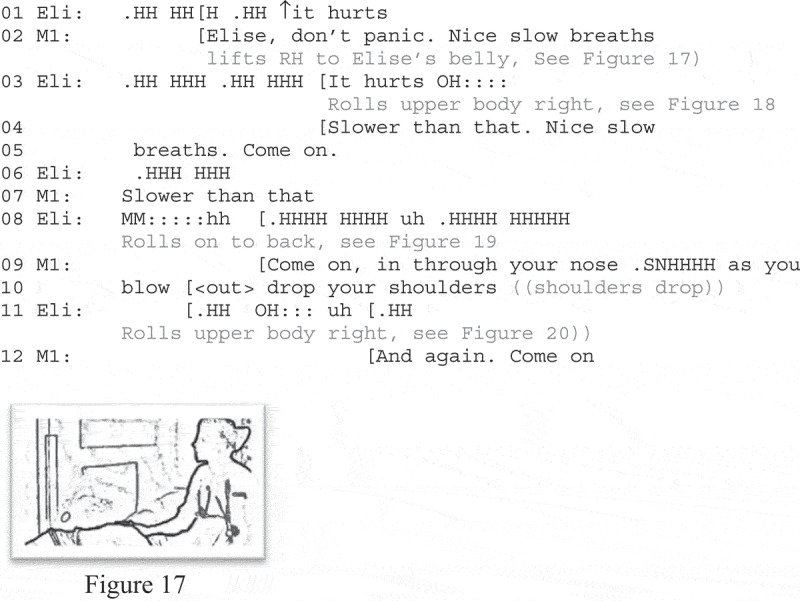

**Extract 11** [VIP02, Elise, Primip, 7:57]: “Nice slow breaths”

**Figure:**
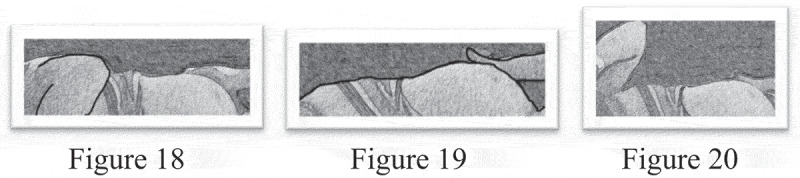

The midwife (M1) treats Elise’s breathing and articulations as displaying “panic” and directs her to produce “nice slow breaths” (line 2). During line 2, M1 places a hand on Elise’s abdomen (Figure 17), likely to facilitate independent evidence of uterine contraction. Her ongoing concern with getting Elise to slow her breathing (lines 4–6) is relevant for managing a contraction.

M1’s focus on breath is prioritized over explicitly responding to Elise’s pain expressions: So, for example, when she says, “nice slow breaths” (lines 2 and 4–5), she does not overtly orient to Elise’s lexical descriptions of “hurt” that precede each of these units. Nevertheless, M1’s recommendations are hearable as instructions for how to breathe to manage (normal, expectable) pain. When Elise does not comply with M1’s directions, and indeed embodies more “panic” via her rapid turns from back to right (notice the presence or absence of Elise’s left arm in Figures 18–20), M1 explicitly describes and models appropriate breathing (lines 9–10). M1 remains active across the full contraction (data not shown), continuing to issue breath-related directives (e.g., “Just control that breathing,” “Really exaggerate that breath”). By directing Elise to breathe in specific ways, M1 is essentially guiding her sensory engagement with the contraction.

## Discussion

This is the first CA study to examine how participants accomplish the non-pathological pain sensations of first-stage labor contractions as a social object through situated practices for its display, recognition, and management. Our analysis contributes to understanding how pain becomes socially organized in medical settings (e.g., Heath, 1989; McArthur, 2018), while also highlighting the distinctiveness of labor as an institutional context. Unlike in primary care, in which participants treat pain displays as diagnostically relevant (Weatherall et al., 2021), participants in our setting orient to pain as an expected, even positive, sign of labor progression. This different orientation is visible in how participants suspend ongoing activities to attend to contractions, treating them as legitimately disrupting, and as having priority over, the normal progressivity of action. Unlike short pain displays (sequentially relevant for ongoing conduct), contractions emerge into and (temporarily) replace ongoing conduct. The midwives’ practices-;particularly their focus on breathing-demonstrate how they support women through pain without attempting to suppress or pathologize it.

Our findings detail key multimodal resources for establishing the onset of the painful sensation of contractions, including breathiness, pain cries, verbal announcements, closing the eyes, and lowering of the head, which together form a multimodal gestalt (Mondada, 2024). These construct contractions as interactionally relevant events that can be both announced by women and noticed by others, highlighting the intersubjective nature of pain displays (Weatherall et al., 2021) and demonstrating midwives’ professional vision (Goodwin, 1994).

Our analysis extends previous CA research on the interactional organization of breathing (e.g., Hoey, 2014; Hofstetter et al., 2021). A key finding is the centrality of breath in socially constituting and managing the pain sensation associated with first-stage contractions. Breathing is a primary resource for establishing pain onset and allows others to recognize and respond to it. Breathing becomes a collective focus during a contraction, shaping the temporality of turns as midwives (and birth partners) offer instructions and assessments of its performance. Importantly, breathing serves dual functions in labor because it both establishes pain and provides a mechanism for managing it. Unlike other medical contexts in which breathing might simply intersubjectively mark pain (Weatherall et al., 2021), in labor it becomes a site of active intervention, with midwives and birth partners collaboratively supporting particular–slow and deep–ways of breathing to help laboring women to manage intense sensations.

Our findings have practical implications for midwifery practice and antenatal education. By detailing how participants convey, recognize, and respond to pain displays in interaction, our analysis identifies practices that organize conduct in the birth room. These insights could be particularly valuable for student midwives developing their professional vision (Goodwin, 1994). Moreover, knowing how breathing becomes collaboratively organized as central to managing pain displays could inform pregnant people how to breathe “well” and how midwives and birth partners can assist them in doing so.

We acknowledge the study’s limitations. The complexity and length of contractions, as well as the novelty of the analytic context, has meant an initial sketch of interactional activities, focusing primarily on contraction onset. Further research is needed to understand the rhythmic quality of breathing, its temporal shaping of recipients’ interaction, and the overall temporal trajectory, including the remission of a contraction and reuptake of suspended activities. Our findings may be specific to the context of British-based midwife-led care. Although our sample was socio-economically diverse, it involved predominantly White participants. This lack of diversity matters, given the proportionately high number of adverse events for laboring people of color (Knight et al., 2019) and harmful stereotypes about their experiences of pain (Henderson et al., 2013). Future research should address barriers to recruiting people of color to pregnancy and labor research.

This study provides a granular account of how the onset of the painful but non-pathological sensations of first-stage labor contractions are constituted in and shape interaction. The article demonstrates the suspensive quality of contractions and the centrality of breath as a resource for both establishing pain and for its management. Our findings, based on a novel analytic context, contribute to a growing understanding of the social organization of the body and sensoriality in interaction.
